# GnRH agonist improves CLBR after one IVF cycle: a propensity score-matched and molecular mechanism study

**DOI:** 10.1530/RAF-25-0045

**Published:** 2025-12-09

**Authors:** Xueqin Cai, Hui Ding, Yi Liu, Wenqian Xiong

**Affiliations:** Department of Obstetrics and Gynecology, Union Hospital, Tongji Medical College, Huazhong University of Science and Technology, Wuhan, China

**Keywords:** cumulative live birth rate (CLBR), GnRH agonist, GnRH antagonist, endometrial receptivity, mesenchymal-epithelial transition

## Abstract

**Graphical Abstract:**

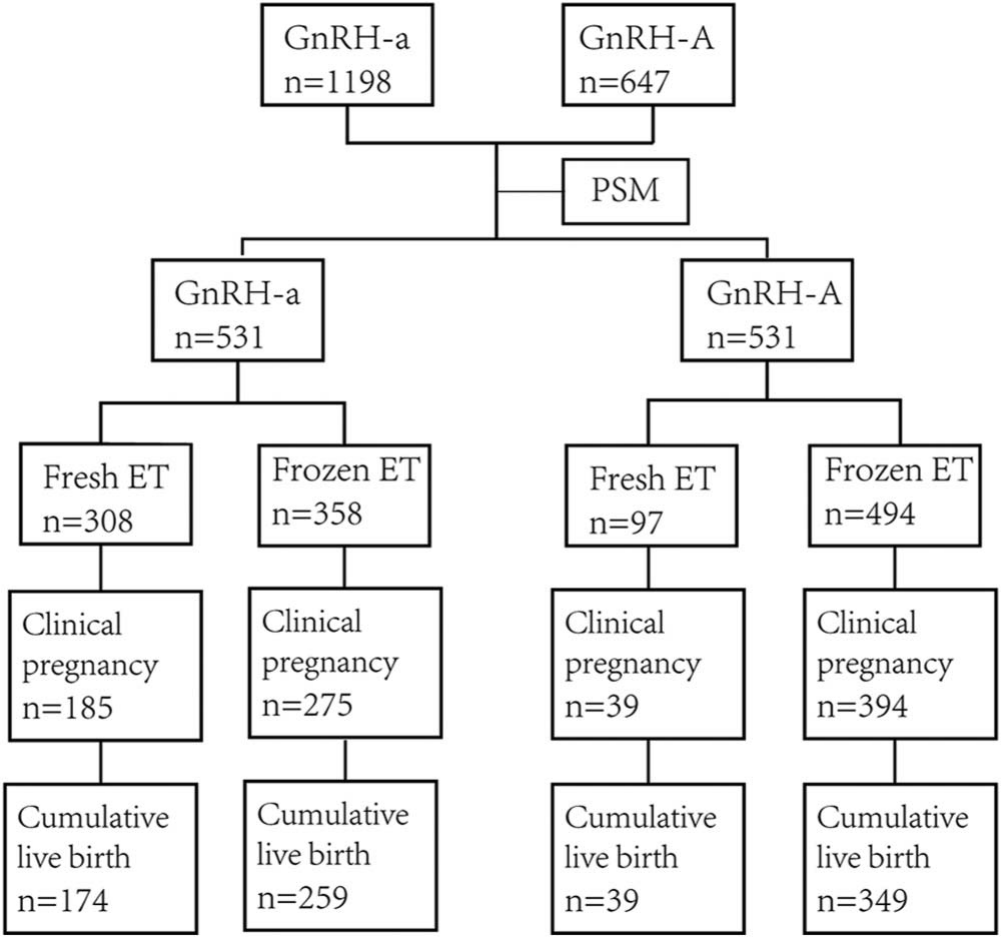

**Abstract:**

The effectiveness of the GnRH antagonist protocol remains controversial due to inconsistent conclusions and inadequate subgroup analyses. The aim of this study was to provide some references for clinicians when choosing the GnRH antagonist protocol for patients. A retrospective cohort study analyzed 1,845 infertility patients aged 20–50 years who underwent IVF/ICSI treatment. They used the GnRH agonist or GnRH antagonist protocol at the Assisted Reproduction Center of Wuhan Union Hospital from June 2023 to June 2024. One-to-one PSM was used to match the population characteristics. The difference of cumulative live birthrates (CLBR) was analyzed by multivariate logistic regression. Endometrial tissues were obtained, and the endometrial receptivity was determined by detecting the expression of mesenchymal–epithelial transition (MET), decidualization markers, and cell implantation *in vitro*. There was a significantly higher CLBR in the GnRH agonist compared with the GnRH antagonist protocol (81.54 versus 73.07%, *P* < 0.05). The GnRH agonist also had a significantly higher clinical pregnancy rate and live birth rate (LBR) per fresh ET cycle compared to the GnRH antagonist. Multivariate logistic regression analysis showed that the ovarian stimulation protocol was an independent risk factor for CLBR. Age and endometrial thickness were significantly correlated with CLBR. Furthermore, the GnRH antagonist reduces endometrial receptivity mainly by hindering the formation of MET in stromal cells and affecting endometrial decidualization. The GnRH antagonist protocol may be associated with inferior LBR per fresh ET cycle and CLBR per cycle compared with the GnRH agonist protocol, likely due to its negative impact on endometrial receptivity.

**Lay summary:**

Doctors are not sure how well one of the methods used for IVF treatment – the GnRH antagonist method – works. It uses drugs to temporarily block brain signals to the ovaries, controlling egg maturation and preventing early egg release (critical for IVF success). It is not known how effective this method is at achieving pregnancy or live birth. We analyzed previous data and lab tests to guide doctors using this method. Results showed that another common method – the GnRH agonist method (using drugs that first boost then lower brain signals to ovaries to control eggs) – had much higher pregnancy rates and LBRs per fresh embryo transfer cycle than the GnRH antagonist one. Data showed three key factors affected the cumulative LBR: the ovarian stimulation plan used, the patient’s age, and the thickness of their womb lining (where embryos attach). Overall, the GnRH antagonist method may lead to lower LBRs (per transfer and per cycle) than the agonist one – most likely because it makes the womb lining less able to support embryos.

## Introduction

Controlled ovarian stimulation (COS), typically involving the co-administration of gonadotropins (Gn) and gonadotropin-releasing hormone (GnRH) analogs, constitutes a cornerstone of *in vitro* fertilization (IVF) treatment. Its primary objective is to generate an optimal number of oocytes, thereby maximizing IVF success rates while prioritizing patient safety. GnRH analogs, encompassing both agonists and antagonists, exert their therapeutic effect by suppressing pituitary gland function. Specifically, they inhibit the endogenous secretion of follicle-stimulating hormone (FSH) and luteinizing hormone (LH), a mechanism critical for preventing premature luteinization, a phenomenon that can compromise oocyte quality and IVF outcomes (Huirne *et al.* 2004, 2005, Weiss *et al.* 2006). Since the 1980s, the long-acting GnRH agonist (GnRH-a) protocol has served as the ‘gold standard’ for COS. Its widespread adoption stems from its proven efficacy in preventing premature LH surges, a key contributor to improved assisted reproductive technology (ART) success metrics (Niederberger *et al.* 2018). Nevertheless, this protocol is associated with notable limitations: it requires an extended treatment duration, correlates with a higher incidence of side effects, and carries an increased risk of ovarian hyperstimulation syndrome (OHSS), a potentially severe iatrogenic complication. The development of GnRH antagonists (GnRH-A) has introduced a valuable alternative for COS. Unlike GnRH agonists, GnRH-A act directly and rapidly to inhibit gonadotropin release, eliminating the need for the initial ‘flare’ phase associated with agonist use (Al-Inany *et al.* 2016, Zhang *et al.* 2021). This unique pharmacodynamic profile confers several advantages to the GnRH-A protocol: a significantly reduced risk of OHSS, a shorter overall treatment timeline, and often a lower required dose of exogenous gonadotropins. These benefits are attributed to the ability of GnRH-A to rapidly block GnRH receptors within hours of administration, enabling more precise COS (Al-Inany *et al.* 2016, Toftager *et al.* 2016, Wang *et al.* 2017).

Despite the long-standing clinical application of GnRH analogs in IVF cycles, a critical controversy persists regarding the relative efficacy of different GnRH analog-based COS protocols. Extensive research has been conducted to compare the performance of GnRH agonist (GnRH-a) and GnRH antagonist (GnRH-A) protocols; however, the findings across these studies remain inconsistent, failing to yield a definitive consensus. Early meta-analyses consistently reported that the clinical pregnancy rate (CPR) associated with GnRH-A protocols was significantly lower than that of GnRH-a protocols, a finding that initially positioned GnRH-a as the preferred option for optimizing pregnancy outcomes (Al-Inany & Aboulghar 2001, Al-Inany *et al.* 2006, Murber *et al.* 2009). In contrast to these earlier observations, subsequent meta-analyses have challenged this conclusion: recent evidence indicates no statistically significant difference in CPRs when directly comparing GnRH-a and GnRH-A COS protocols. This shift in findings suggests that the efficacy gap once perceived between the two protocols may be narrower than previously thought or even non-existent under certain clinical conditions (Al-Inany *et al.* 2011, Depalo *et al.* 2012, Wang *et al.* 2017, Liu *et al.* 2023).

The cumulative live birth rate (CLBR) is defined as the percentage of patients who achieve a live birth following the initiation of ovarian stimulation. Notably, this metric accounts for both fresh embryo transfer (FET) cycles and frozen embryo transfer cycles, integrating outcomes across the entire spectrum of embryo utilization after COS. As such, CLBR serves as a definitive endpoint for assessing the effectiveness of a complete ART treatment cycle, as it reflects success from the onset of ovarian stimulation through the first successful live delivery (Harbin Consensus Conference Workshop Group; *et al.* 2014, Maheshwari *et al.* 2015, Kim 2023). In a key investigation, Yang and colleagues explored CLBR disparities between GnRH agonist (GnRH-a) and GnRH antagonist (GnRH-A) protocols across patient subgroups. Their findings revealed that among patients with suboptimal ovarian response (SOR), the CLBR was significantly higher in the GnRH-a protocol group compared to the GnRH-A protocol group. In contrast, no statistically significant difference in CLBR was observed between the two protocols in patients without SOR (Yang *et al.* 2021).

Therefore, the present study aimed to address three key objectives: first, it sought to compare the CLBR between the GnRH agonist (GnRH-a) and GnRH antagonist (GnRH-A) protocols, stratified by the number of retrieved oocytes, following one complete ART cycle in which all available embryos were utilized. Second, to validate the clinical outcomes of these two protocols, an internal retrospective cohort study was conducted using data from our reproductive medicine center, supplemented with propensity-score matching (PSM) analysis to minimize confounding biases. Third, the study aimed to further clarify the effects of the two protocols on endometrial receptivity through an investigation of underlying molecular mechanisms. Collectively, these efforts were intended to provide clinicians with evidence-based references for selecting the optimal COS protocol for individual patients, thereby enabling more accurate prediction of live birth probabilities.

## Materials and methods

### Ethical approval

All protocols, data collection, and storage procedures involving human participants in this study were approved by the Medical Ethics Committee of Tongji College, Huazhong University of Science and Technology (Approval No.: 2022-S167) and conducted in compliance with the 1964 Declaration of Helsinki and its subsequent amendments. Investigators were required to obtain informed consent before enrolling participants in the clinical trial. Included patients were those who received either the GnRH-a or GnRH-A protocol and met all the following inclusion criteria: aged 20–50 years, with indications for IVF or intracytoplasmic sperm injection (ICSI). Exclusion criteria were as follows: i) chromosome abnormalities in either spouse or requirement for preimplantation genetic testing (PGT) for assisted conception; ii) moderate or severe endometriosis; iii) use of donor oocytes or sperm; iv) abnormal uterine cavity confirmed by hysterosalpingogram or hysteroscopy; v) transfer of embryos derived from two different stimulated cycles; vi) history of oocyte activation or oocyte freezing cycles. All women were followed up via telephone until pregnancy outcomes were confirmed.

### Ovarian stimulation

Women underwent ovarian stimulation using either the GnRH-a or GnRH-A protocol. For the GnRH-a protocol, pituitary downregulation was achieved via daily subcutaneous injection of 0.1 mg GnRH-a (Triptorelin acetate: 0.1 mg, Switzerland) initiated during the mid-luteal phase (cycle days 21–23) of the menstrual cycle preceding treatment. After 20 days of downregulation, ultrasound and serum hormone testing were performed to confirm the following criteria: endometrial thickness ≤5 mm, follicle diameter 5–7 mm, serum estradiol (E2) < 50 pg/mL, progesterone (P) < 1 ng/mL, and LH < 1 mIU/mL. Ovarian stimulation was then initiated with human menopausal gonadotropin (HMG, Lebaode, Lizhu, China) or FSH (Urofollitropin for Injection, Lizhu) at a daily dose of 150–225 U. The Gn dose and follow-up frequency were adjusted based on follicular development and serum hormone levels. For the GnRH-A protocol, ovarian stimulation was initiated on cycle days 2–3 of the menstrual cycle, following the same procedure as the GnRH-a protocol. On the 6th day of stimulation, GnRH-A (Ganirelix acetate: 0.25 mg, Switzerland) was added at 0.25 mg daily, continuing until the trigger day. When ≥1 follicles reached a diameter of ≥18 mm or ≥3 follicles reached ≥16 mm, human chorionic gonadotropin (hCG: 6,000–10,000 U, Lizhu) was administered to induce final oocyte maturation. Oocyte retrieval was performed 36 h after hCG injection.

### Fertilization and embryo evaluation

Insemination was performed approximately 2 h after each oocyte retrieval, using 20,000–30,000 motile sperm per oocyte. Intracytoplasmic sperm injection (ICSI) was conducted if the total number of motile sperm post-washing was <10^5^ or the percentage of morphologically normal sperm was <1%. Fertilization was confirmed by assessing oocytes for the presence of two pronuclei. On day 3 post-oocyte retrieval, embryos were graded 1–6 based on blastomere evenness and fragmentation rate, in accordance with previously established criteria (Veeck 1988, Rienzi *et al.* 2005). Embryos with 6–8 cells and a grade of 1 or 2 were defined as good-quality embryos (Anagnostopoulou *et al.* 2022). Selected low-quality embryos were subjected to extended culture until reaching the blastocyst stage.

### Fresh and frozen embryo transfer

In fresh embryo transfer cycles, a maximum of two day-3 embryos (post-retrieval) were transferred under transabdominal ultrasound guidance. Remaining good-quality day-3 embryos, as well as morphologically intact day-5 or day-6 blastocysts, were cryopreserved via vitrification. For patients who failed to achieve pregnancy in the fresh transfer cycle or required delayed fresh transfer, frozen embryo transfer (FET) was performed at least 2 months after the ovarian stimulation cycle. Following embryo thawing, embryos were placed in culture dishes for viability assessment and further development. Only embryos with >50% viable blastomeres post-thaw were eligible for transfer in FET cycles. A maximum of two embryos (or blastocysts) were transferred per FET cycle.

### Outcomes measures

The primary outcome measure was the CLBR, defined as the delivery of at least one live newborn at ≥22 weeks of gestation following ovarian stimulation, along with the time to live birth. The secondary indicators were clinical indicators and laboratory indicators. Clinical indicators included total Gn use, duration of Gn administration, serum estradiol (E2) level on the hCG trigger day, endometrial thickness, incidence of moderate-to-severe ovarian hyperstimulation syndrome (OHSS), CPR, chemical pregnancy rate, miscarriage rate, and multiple pregnancy rate. Laboratory indicators included number of oocytes retrieved, number of mature oocytes, number of fertilized oocytes (and fertilization rate), number of cleaved embryos (and cleavage rate), number of cryopreserved embryos, number of good-quality embryos, and number of blastocysts formed.

### Patient and tissue sample collection

Normal proliferative-phase endometrial tissues were collected from 15 women with regular menstrual cycles whose infertility was attributed to tubal factors, for subsequent cell experiments. Secretory-phase endometrial tissues were obtained from 12 women who underwent ovarian stimulation via either the GnRH-a (*n* = 6) or GnRH-A (*n* = 6) protocol. These 12 patients had abandoned fresh embryo transfer due to unsuitable endometrial conditions for transfer or other personal reasons, and all embryos were cryopreserved on the scheduled transfer day. All secretory-phase endometrial samples were used for immunohistochemical staining.

### Immunohistochemical (IHC) staining

Endometrial tissues from patients receiving the GnRH-a (long protocol) and GnRH-A (antagonist protocol) were immediately fixed in 4% paraformaldehyde. Subsequent procedures, including paraffin embedding, paraffin sectioning, and immunohistochemical (IHC) staining, were performed by Biossci Biotechnology Co., Ltd (China). Primary antibodies targeting Vimentin and E-cadherin were used for IHC staining. Data were ultimately analyzed and processed using ImageJ software. Detailed information about the antibodies is available in Supplementary Table 1 (see section on Supplementary materials given at the end of the article).

### Cell culture and drug treatment

The protocol for primary culture of human endometrial stromal cells (HESCs) was performed as previously described (Xiong *et al.* 2024). ESC purity was verified via cellular immunohistochemistry and immunofluorescence (Xiong *et al.* 2024). Cells were cultured in DMEM/F12 medium supplemented with 10% fetal bovine serum (FBS) and maintained in a humidified incubator with 5% CO_2_ at 37°C.

GnRH-a (Triptorelin acetate: 0.1 mg, Switzerland) and GnRH-A (Ganirelix acetate: 0.25 mg, Switzerland) were used for cell experiments. Based on drug metabolism and serum concentration stability, the test concentrations of GnRH-a were set at 5.00 × 10^−10^ mol/L, 9.33 × 10^−10^ mol/L, and 2.04 × 10^−10^ mol/L, while those of GnRH-A were set at 3.55 × 10^−10^ mol/L and 8.87 × 10^−10^ mol/L. The final working concentrations, 5.00 × 10^−10^ mol/L for GnRH-a and 3.55 × 10^−10^ mol/L for GnRH-A, were determined by detecting the expression of decidualization markers (prolactin and insulin-like growth factor binding protein) and mesenchymal–epithelial transition (MET) markers (E-cadherin and Vimentin) (Supplementary Fig. 1).

### Decidualization *in vitro*

When the cell density reached 60%, cells were pretreated with the optimal concentrations of GnRH-a (5.00 × 10^−10^ mol/L) and GnRH-A (3.55 × 10^−10^ mol/L), respectively. Subsequently, the culture medium was replaced with phenol red-free DMEM/F12 medium supplemented with 2% FBS. To induce cellular decidualization, medroxyprogesterone acetate (MPA, 1 μM, MCE, HY-B0469S) and 8-bromo-cyclic adenosine monophosphate (8-Br-cAMP, 0.5 mM, Selleck, S7857) were added, and the induction was maintained for 6 days. Cellular decidualization was evaluated by detecting the expression of decidualization markers and observing changes in cell morphology.

### RNA extraction, reverse transcription, and real-time PCR

Total cellular RNA was extracted from all samples using TRIzol reagent (Vazyme, China) following the manufacturer’s instructions. The extracted RNA was then reverse-transcribed into complementary DNA (cDNA) using the HiScript III First Strand cDNA Synthesis Kit (Vazyme). Reverse transcription-quantitative polymerase chain reaction (RT-qPCR) was performed using 2× Green ® Master qPCR Mix (containing SYBR Green I and UDG; Qingke, China). The sequences of the primers used are provided in Supplementary Table 1. The relative expression levels of target genes were calculated using the △△Ct method.

### Western blot analysis

Proteins were extracted using radioimmunoprecipitation assay (RIPA) buffer (NCM Biotech, China) supplemented with phenylmethylsulfonyl fluoride (PMSF, Servicebio, China). The protein concentration was determined and quantified using a BCA Protein Assay Kit (Vazyme, China). Equal amounts of protein (30 μg per sample) were separated by 10% sodium dodecyl sulfate-polyacrylamide gel electrophoresis (SDS-PAGE) and then transferred onto polyvinylidene fluoride (PVDF) membranes (Millipore, USA). After blocking with 5% non-fat milk in Tris-buffered saline containing 0.1% Tween-20 (TBST) for 1 h at room temperature, the membranes were incubated with primary antibodies overnight at 4°C. Following washing with TBST, the membranes were incubated with secondary antibodies for 1 h at room temperature. After an additional round of TBST wash, protein bands on the membranes were visualized using an enhanced chemiluminescence (ECL) detection reagent (Biology, Wuhan, China) under a chemiluminescence detection system. The gray values of the protein bands were analyzed using ImageJ software. Detailed information about the antibodies is available in Supplementary Table 2.

### Enzyme-linked immunosorbent assay

After cells underwent drug pretreatment and *in vitro* decidualization, cell culture supernatants were collected and centrifuged to remove cell debris. Commercially available enzyme-linked immunosorbent assay (ELISA) kits were used for detection: a prolactin (PRL) ELISA kit (Ruixinbio, China; cat. no. RX106036H) and an insulin-like growth factor binding protein 1 (IGFBP1) ELISA kit (Ruixinbio, China; Cat. No. RX104919H) were employed to measure the levels of PRL and IGFBP1, respectively. All samples were assayed in duplicate. The concentrations of PRL and IGFBP1 were expressed as mIU/mL and ng/mL of cell supernatant, respectively.

### Phalloidin staining

Cells were fixed with 4% paraformaldehyde for 10 min, rinsed with phosphate-buffered saline (PBS), and then permeabilized with 0.5% Triton X-100 for 5 min. After an additional PBS rinse, cells were stained with a prepared TRITC-labeled phalloidin working solution (cat. no. 40734ES75, Yeasen Biotechnology, China) and incubated at room temperature for 30 min in the darkness. Following another PBS wash, cell nuclei were counterstained with 100 nM DAPI solution (cat. no. BL120A, Biosharp, China) for 30 s, and cells were rinsed again with PBS. The samples were mounted using fluorescent mounting medium, observed under a confocal laser scanning microscope, and stored at 4°C in the darkness.

### Cell implantation *in vitro*

HTR-8/SVneo cells (an immortalized cell line derived from early pregnancy villous explants) were co-cultured with decidualized monolayer HESCs to simulate embryo implantation (Ojosnegros *et al.* 2021). First, single-cell suspensions of HTR-8/SVneo cells were seeded into low-adhesion 96-well plates at a density of approximately 5,000 cells per well. The culture medium was replaced with RPMI-1640 medium supplemented with 5% FBS and 2 mg/mL methylcellulose. After 72 h of culture, HTR-8/SVneo multicellular spheres were formed; spheres with a diameter of 70–100 μm were selected using a cell strainer. These HTR-8/SVneo spheres were then transferred onto decidualized monolayer HESCs. Following 12 h of co-culture at 37°C, the cells were rinsed with sterile PBS to remove non-adherent spheres. Adherent spheres were counted under a light microscope, and the spheroid adhesion rate was calculated as the percentage of adherent spheres relative to the total number of spheres initially transferred.

### Statistical analysis

Data analyses were performed using GraphPad Prism 8.0 and SPSS 26.0 software. Continuous variables with a normal distribution are expressed as mean ± standard deviation (x̄ ± SD), and categorical variables as proportions or rates (%). Propensity score matching (PSM) was used to mitigate bias from observed variables and confounding factors affecting treatment outcomes. A 1:1 nearest-neighbor matching approach was applied, with nine variables included: maternal age, body mass index (BMI), stimulation cycle type, antral follicle count (AFC), infertility type, baseline FSH, anti-Müllerian hormone (AMH), infertility etiology, and insemination method. For between-group comparisons, unpaired Student’s *t*-test or Mann–Whitney U test was used based on data normality. Comparisons of continuous variables across multiple groups were performed using one-way ANOVA followed by least significant difference post-hoc tests. Categorical data were compared using the chi-square test or Fisher’s exact test, as appropriate. Binary logistic regression was used to identify factors influencing CLBR. Results are presented as mean ± SD. A two-tailed *P* < 0.05 was considered statistically significant.

## Result

### Baseline characteristics before and after matching

Baseline characteristics before matching are presented in Table 1. Significant differences were observed between groups in maternal age, BMI, primary infertility status, number of cycles, AFC, baseline FSH, AMH, and infertility etiology (*P* < 0.05), with the exception of insemination method. To eliminate bias arising from uneven baseline characteristics, all cycles were matched 1:1 using PSM based on the aforementioned nine variables, resulting in a highly comparable control group. After PSM, 531 couples remained in each group. Post-matching baseline demographic characteristics are also shown in Table 1, with no significant differences observed between the two groups for any of the baseline variables (*P* > 0.05).

**Table 1:** Comparison of baseline characteristics between the two groups before and after PSM. Data are presented as *n* (%), mean SD, or as median (IQR).

Characteristics	Before PSM			After PSM		
	GnRH-a	GnRH-A	*P*-value	GnRH-a	GnRH-A	*P*-value
Total *n*	1,198	647		531	531	
Maternal age, years	30.61 ± 3.47	31.56 ± 4.56	<0.0001*	31.01 ± 3.58	30.83 ± 4.04	0.4309
BMI, Kg/m^2^	22.74 ± 3.80	23.27 ± 3.84	0.046*	23.36 ± 4.17	23.18 ± 3.73	0.4769
Primary infertility	646 (53.92%)	335 (51.78%)	0.3794	285 (53.67%)	284 (53.48%)	0.9509
First stimulation cycle						
Yes	1,077 (89.90%)	494 (76.35%)	<0.0001*	449 (84.56%)	434 (81.73%)	0.2198
No	121 (10.10%)	153 (23.65%)		82 (15.44%)	97 (18.27%)	
Antral follicle count	12.00 (9.00, 15.00)	10.00 (7.00, 14.00)	<0.0001*	11.00 (8.00, 14.00)	10.00 (7.00, 14.00)	0.3420
Basal FSH (IU/L)	6.64 (5.82, 7.69)	6.88 (5.77, 8.22)	0.004	6.60 (5.85, 7.83)	6.80 (5.72, 8.09)	0.6250
AMH (ng/mL)	4.76 ± 3.11	5.35 ± 4.31	0.002	5.30 ± 3.78	5.28 ± 3.95	0.9380
Infertility factors						
Tubal factor	503 (41.99%)	205 (31.68%)	<0.0001*	179 (33.71%)	191 (35.97%)	0.8779
Male factor	111 (9.27%)	40 (6.18%)		37 (6.97%)	38 (7.16%)	
Ovulation disorder	171 (14.27%)	144 (22.26%)		125 (23.54%)	113 (21.28%)	
Unexplained	267 (22.29%)	98 (15.15%)		88 (16.57%)	86 (16.20%)	
Diminished ovarian reserve	37 (3.09%)	60 (9.27%)		28 (5.27%)	34 (6.40%)	
Mixed factors	109 (9.10%)	100 (15.46%)		74 (13.94%)	69 (12.99%)	
Insemination method						
IVF	847 (70.70%)	430 (66.46%)	0.02393	356 (67.07%)	354 (66.67%)	0.7440
ICSI	281 (23.46%)	173 (26.74%)		144 (27.12%)	140 (26.74%)	
IVF + ICSI	1 (0.08%)	0 (0)		0 (0)	0 (0)	
Rescue ICSI	69 (5.76%)	44 (6.80%)		31 (5.84%)	37 (6.97%)	

PSM, propensity score-matched; BMI, body mass index; FSH, follicle-stimulating hormone; AMH, anti-Müllerian hormone; ICSI, intracytoplasmic sperm injection; IVF, *in vitro* fertilization.

* *P* < 0.05.

### Comparison of clinical and laboratory indexes of GnRH-a and GnRH-A protocols

The total Gn dosage was comparable between the two protocols. However, the GnRH-A protocol was associated with a shorter duration of ovarian stimulation and significantly lower serum estradiol (E2) levels on the hCG trigger day compared with the GnRH-a protocol. No significant differences were observed between the two protocols in terms of the number of oocytes retrieved, number of mature oocytes, number of fertilized oocytes, number of cleaved embryos, fertilization rate, cleavage rate, number of cryopreserved embryos, number of good-quality embryos, or number of transferable embryos (Table 2).

**Table 2:** Comparison of clinical and laboratory indicators and treatment outcomes between the GnRH-a protocol and the GnRH-A protocol after propensity score matching.

	GnRH-a (*n* = 531)	GnRH-A (*n* = 531)	*P*-value
Dosage of Gn (IU)	2,214.78 (600.00, 7,050.00)	2,124.33 (900.00, 6,000.00)	0.0772
Duration of Gn (days)	9.45 (3.00, 17.00)	9.21 (5.00, 15.00)	0.0119*
Serum estradiol level on trigger day	2,988.00 (2,119.61, 4,250.67)	2,393.53.00 (1,429.61, 3,962.00)	<0.0001*
No. of oocytes retrieved	14.97 (3.00, 71.00)	14.66 (1.00, 74.00)	0.5476
No. of mature oocytes	12.10 (0.00, 53.00)	11.76 (0.00, 55.00)	0.4429
No. of oocytes fertilized	8.32 (0.00, 42.00)	8.07 (0.00, 40.00)	0.4511
Fertilization rate	4,418/7,951 (55.57%)	4,285/7,782 (55.06%)	0.5317
No. of embryos cleaved	8.16 (0.00, 40.00)	7.82 (0.00, 42.00)	0.3727
Cleavage rate	4,334/5,732 (75.16%)	4,180/5,609 (74.52%)	0.1854
No. of embryos frozen	4.26 (0.00, 21.00)	4.48 (0.00, 42.00)	0.3159
No. of top-quality embryos	3.18 (0.00, 21.00)	3.05 (0.00, 24.00)	0.3727
No. of blastocysts formed	3.72 (0.00, 19.00)	3.88 (0.00, 22.00)	0.4680

Gn, gonadotropins.

* *P* < 0.05.

### Comparison of treatment outcome of GnRH-a and GnRH-A protocols

A total of 308 fresh embryo transfer (ET) cycles and 358 frozen ET cycles were conducted in the GnRH-a protocol, while 97 fresh ET cycles and 494 frozen ET cycles were performed in the GnRH-A protocol. The total number of embryos transferred across all cycles was comparable between the two protocols, yet significant differences in the number of embryos transferred were observed in fresh and frozen ET cycles separately: in fresh ET cycles, 36.36% of cycles in the GnRH-a protocol involved transferring two embryos, which was significantly lower than the 53.61% in the GnRH-A protocol; in frozen ET cycles, 54.19% of cycles in the GnRH-a protocol transferred two embryos, significantly higher than the 41.7% in the GnRH-A protocol. In fresh ET cycles, the GnRH-a protocol exhibited significantly greater endometrial thickness, higher CPR per cycle, higher implantation rate, and higher live birth rate per transfer cycle compared to the GnRH-A protocol, with no significant differences in chemical pregnancy rate per cycle, miscarriage rate, or multiple pregnancy rate. In frozen ET cycles, only the multiple pregnancy rate differed significantly between the two protocols, while no significant differences were found in other indicators, such as endometrial thickness and various pregnancy rates. Across all ET cycles, the GnRH-A protocol had a significantly higher CPR per cycle, but there were no significant differences in endometrial thickness, incidence of moderate-to-severe ovarian hyperstimulation syndrome (OHSS), and other indicators. In contrast, the GnRH-a protocol showed significantly higher CPR per woman and live birth rate per woman (Table 3).

**Table 3:** Comparison of pregnancy outcomes between the GnRH-a group and the GnRH-A group after propensity score matching.

Characteristics	GnRH-a			GnRH-A			*P*-value		
	Fresh-ET	Frozen-ET	Total	Fresh-ET	Frozen-ET	Total	1	2	3
No. of embryo transfer cycles	308	358	666	97	494	591			
No. of embryos transferred									
1	196/308 (63.64%)	164/358 (45.81%)	360/666 (54.05%)	45/97 (46.39%)	288/494 (58.30%)	333/591 (56.35%)	0.0030*	0.0004	0.4267
2	112/308 (36.36%)	194/358 (54.19%)	306/666 (45.95%)	52/97 (53.61%)	206/494 (41.70%)	258/591 (43.65%)			
Endometrial thickness (mm)	11.12 (6.00, 20.00)	10.54 (6.00, 19.00)	10.81 (6.00, 20.00)	10.57 (6.00, 16.00)	10.66 (4.50, 19.00)	10.65 (4.50, 19.00)	0.0417*	0.4119	0.1802
Rate of moderate or severe OHSS	-	-	16/531 (3.01%)	-	-	20/531 (3.77%)	-	-	0.4980
CH-PR/ cycle	21/308 (6.08%)	24/358 (6.70%)	45/666 (6.76%)	11/97 (11.34%)	26/494 (5.26%)	37/591 (6.26%)	0.1931	0.3771	0.7222
CL-PR/ cycle	185/308 (60.06%)	283/358 (79.05%)	468/666 (70.27%)	39/97 (40.21%)	407/494 (82.39%)	446/591 (75.47%)	0.0007*	0.2502	0.0424*
Implantation rate	212/420 (50.48%)	342/552 (61.96%)	554/972 (57.00%)	49/149 (32.89%)	468/700 (66.86%)	517/849 (60.90%)	0.0003*	0.0742	0.0917
Miscarriage rate	9/185 (4.86%)	14/282 (4.96%)	33/467 (7.07%)	0/39 (0)	21/407 (5.16%)	21/446 (12.56%)	0.3653	0.9999	0.9999
MPR	18/185 (9.73%)	60/282 (21.28%)	78/467 (16.70%)	2/39 (5.13%)	61/407 (14.99%)	63/446 (14.13%)	0.5395	0.0329*	0.2815
LBR/ transfer cycle	174/308 (56.49%)	260/368 (72.63%)	434/666 (65.17%)	39/97 (40.21%)	351/494 (71.05%)	390/591 (65.99)	0.0071*	0.6148	0.7665
CH-PR/ woman	21/531 (3.95%)	24/531 (4.52%)	45/531 (8.47%)	11/531 (2.07%)	26/531 (4.90%)	37/531 (6.97%)	-	-	0.4211
CL-PR/ woman	185/531 (34.84%)	275/531 (51.79%)	460/531 (86.63%)	39/531 (7.31%)	394/531 (74.20%)	433/531 (81.54%)	-	-	0.0290*
LBR/ woman	174/531 (32.77%)	259/531 (48.76%)	433/531 (81.54%)	39.531 (7.34%)	349/531 (65.73%)	388/531 (73.07%)	-	-	0.0012*

CH-PR, chemical pregnancy rate; CL-PR, clinical pregnancy rate; MPR, multiple pregnancy rate; LBR, live birth rate; OHSS, ovarian hyperstimulation syndrome; ET, embryo transfer; LBR per woman = cumulative live birth rate.

* *P* < 0.05.

### Logistic regression analysis of multiple factors influencing CLBR

After adjusting for confounding factors (including female age, number of oocytes retrieved, infertility etiology, AFC, insemination method, BMI, baseline FSH, AMH, total Gn dosage, and endometrial thickness), the CLBR per oocyte retrieval cycle was significantly lower in the GnRH-A protocol compared with the GnRH-a protocol (odds ratio (OR) = 0.63, 95% confidence interval (CI): 0.464–0.856, *P* = 0.030). As shown in Table 4, female age and endometrial thickness were significantly correlated with CLBR (both *P* < 0.05).

**Table 4:** Binary logistic regression analysis of factors for prediction of cumulative live birth rate from the fresh plus all frozen-thawed transfer cycles combined after the same index stimulation cycle.

Characteristic/covariate strata	OR	95% CI	*P*
Age of women (years)			
≤35	Ref		
36–39	0.409	0.259–0.646	<0.001*
≥40	0.068	0.019–0.243	<0.001*
Cycle number			
First	Ref		
Repeated	1.057	0.690–1.620	0.799
Treatment protocol			
GnRH-a	Ref		
GnRH-A	0.630	0.464–0.856	0.030*
Causes of infertility			
Tubal	Ref		
Male factor	1.315	0.646–2.679	0.451
Anovulatory	1.458	0.860–2.472	0.161
Unexplained	0.825	0.538–1.265	0.378
DOR	1.666	0.822–3.375	0.156
Mixed factors	1.143	0.670–1.951	0.623
Antral follicle count			
0–5	Ref		
6–15	1.412	0.867–2.298	0.166
≥16	1.605	0.810–3.182	0.175
Insemination method			
IVF	Ref		
ICSI	0.979	0.653–1.469	0.920
IVF + RICSI	0.732	0.407–1.317	0.297
Body mass index	0.975	0.936–1.015	0.222
Basal FSH level	0.993	0.921–1.072	0.865
Basal AMH level	0.993	0.972–1.102	0.289
Total dose of Gn used	1.000	1.000–1.000	0.787
Endometrial thickness	1.114	1.034–1.200	0.040*

Ref, reference group; CI, confidence interval; FSH, follicle-stimulating hormone; ICSI, intracytoplasmic sperm injection; IVF, *in vitro* fertilization; RICSI, rescue intracytoplasmic sperm injection; DOR, diminished ovarian reserve.

* *P* < 0.05.

### Expression of MET biomarkers in the secretory endometrium in GnRH-a and GnRH-A protocol

**Figure 1:**
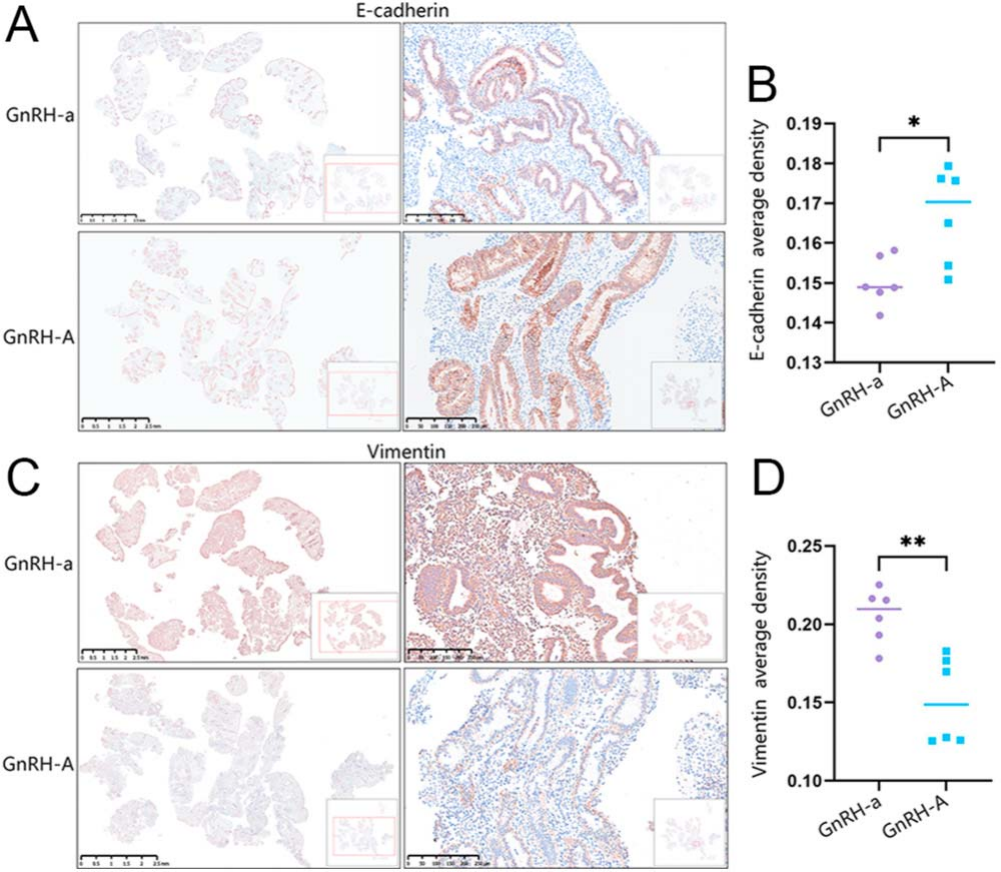
Expression of MET biomarkers in secretory-phase endometrium of the GnRH-a and GnRH-A group. (A) Representative photomicrographs of E-cadherin and Vimentin expression in the secretory-phase endometrium of the GnRH-a group. (B) Representative photomicrographs of E-cadherin and Vimentin expression in the secretory-phase endometrium of the GnRH-A group. (*P < 0.05, **P < 0.01, GnRH-a compared with GnRH-A group; GnRH-a = 6, GnRH-A = 6).

Immunohistochemical (IHC) analysis examined the expression of MET markers in secretory-phase endometrium from GnRH-a and GnRH-A protocols (Fig. 1A and C), with average biomarker densities summarized in Fig. 1B and D. Compared to the GnRH-a protocol, E-cadherin (expressed exclusively in epithelial cells) showed increased expression in the GnRH-A protocol, while Vimentin (expressed in both epithelial and stromal cells) was significantly lower in the GnRH-A protocol, indicating MET impairment in the secretory endometrium of the GnRH-A protocol.

### Effect of GnRH-A on decidualization of ESC *in vitro*

**Figure 2:**
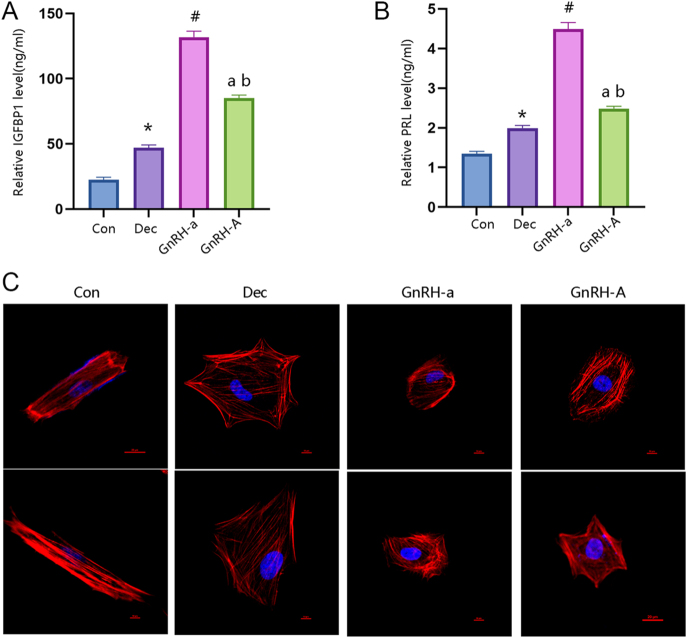
GnRH-A was downregulated during the decidualization of primary endometrial stromal cells (ESCs) *in vitro*. (A, B, C, D) ESCs were divided into four groups (Con, Dec, GnRH-a, and GnRH-A) *in vitro*. (A and B) Protein levels of IGFBP1 and PRL were examined by ELISA. (C and D) Representative Western blot analysis showing the levels of E-cadherin and Vimentin. (Con = control; Dec = decidualization; *P < 0.05, Dec compared with Con group; ^#^*P* < 0.05, GnRH-a compared with Dec group; ^a^P<0.05, GnRH-A compared with Dec group; ^b^P<0.05, GnRH-A compared with GnRH-a group).

**Figure 3:**
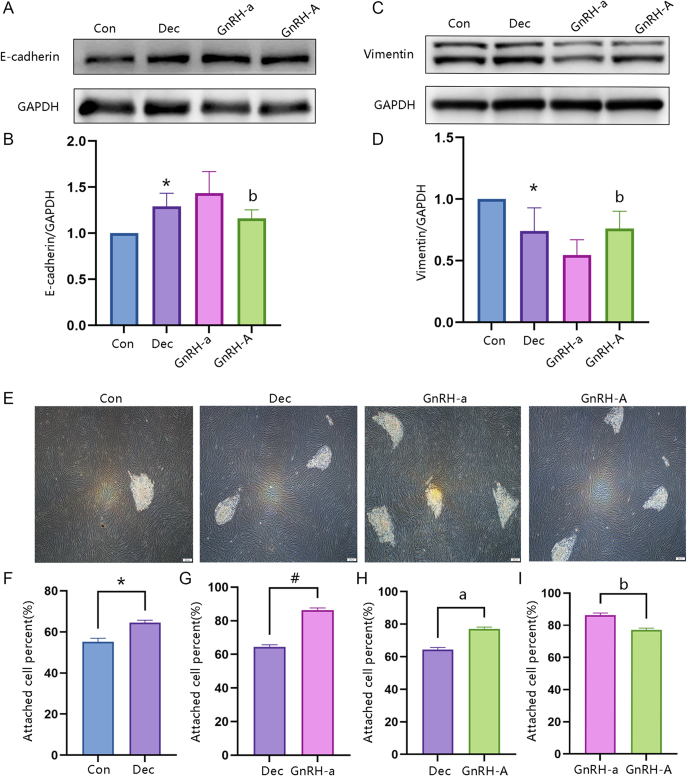
GnRH-A was involved in regulating cellular decidualization and embryo implantation. (A and B) ESCs were divided into four groups (Con, Dec, GnRH-a, and GnRH-A) *in vitro*. (A) Cytoskeleton staining to determine the cellular morphology of ESCs. (B) Representative images and adhesion rate were presented to show adhered HTR8 spheroids attached to ESCs. (Con = control; Dec = decidualization; *P < 0.05, Dec compared with Con group; ^#^*P* < 0.05, GnRH-a compared with Dec group; ^a^P<0.05, GnRH-A compared with Dec group; ^b^P<0.05, GnRH-A compared with GnRH-a group).

ELISA was used to investigate the effects of GnRH-a and GnRH-A on decidualization of ESC. The relative protein levels of IGFBP1 and PRL were upregulated following decidualization (Fig. 2A and B). Both GnRH-a and GnRH-A were able to increase the protein expression of IGFBP1 and PRL compared with the decidualization group. Meanwhile, further analysis found significantly reduced expression of IGFBP1 and PRL in the GnRH-A group compared with the GnRH-a group. As shown in the results of immunofluorescence staining, a transition in ESC morphology from elongated spindle-like cells to round-shaped cells after decidualization. The cell morphology of both GnRH-a and GnRH-A changed from elongated spindle-like cells to round-shaped cells (Fig. 2C). Western blot analysis demonstrated that the E-cadherin protein level increased in the decidualization group compared to the control, whereas Vimentin protein levels decreased. Compared with the decidualization group, both GnRH-a and GnRH-A were able to increase the protein expression of E-cadherin while decreasing the expression of Vimentin. Further analysis found reduced expression of E-cadherin and increased expression of Vimentin in the GnRH-A compared with the GnRH-a group (Fig. 3A, B, C, D). Functional analysis using spheroid adhesion assays on both *in vitro* models revealed that the decidualization group significantly enhanced stromal cell adhesion to HTR8/SVneo spheroids compared with the control group. Both GnRH-a and GnRH-A were able to increase stromal cell adhesion compared with the decidualization group. Further analysis found impaired stromal cell adhesion in the GnRH-A group compared with the GnRH-a group (Fig. 3E, F, G, H, I). Collectively, these findings suggest that GnRH-A impaired decidualization of HESCs by reducing MET formation in HESCs.

ELISA was used to investigate the effects of GnRH-a and GnRH-A on ESC decidualization: relative protein levels of IGFBP1 and PRL were upregulated after decidualization (Fig. 2A and B); both GnRH-a and GnRH-A increased IGFBP1 and PRL protein expression compared with the decidualization group, though IGFBP1 and PRL expression was significantly lower in the GnRH-A group than in the GnRH-a group. IHC staining showed that HESCs transitioned from elongated spindle-like cells to round-shaped cells after decidualization, with both GnRH-a and GnRH-A groups also exhibiting this morphological transition (Fig. 2C). Western blot analysis revealed that compared with the control group, the decidualization group had increased E-cadherin protein levels and decreased Vimentin protein levels; relative to the decidualization group, both GnRH-a and GnRH-A upregulated E-cadherin and downregulated Vimentin, while the GnRH-A group showed lower E-cadherin and higher Vimentin expression than the GnRH-a group (Fig. 3A, B, C, D). Spheroid adhesion assays in *in vitro* models demonstrated that the decidualization group significantly enhanced stromal cell adhesion to HTR8/SVneo spheroids compared with the control group; both GnRH-a and GnRH-A improved stromal cell adhesion relative to the decidualization group, but adhesion was impaired in the GnRH-A group compared with the GnRH-a group (Fig. 3E, F, G, H, I). Collectively, these findings suggest that GnRH-A impairs ESC decidualization by reducing MET formation in HESCs.

## Discussion

COS is a critical component of *in vitro* fertilization/intracytoplasmic sperm injection (IVF/ICSI), aiming to retrieve a sufficient number of oocytes in a single cycle. It primarily relies on two protocols: the GnRH-a protocol and the GnRH-A protocol. However, the efficacy of the GnRH-A protocol remains controversial due to inconsistent research conclusions and insufficient subgroup analyses. Given the ongoing debates surrounding reproductive outcomes with these two regimens, the present study analyzed clinical data to compare the clinical outcomes of the GnRH-a and GnRH-A protocols for COS in IVF treatment. As a retrospective study, it was inevitable that certain confounding factors might influence the results. Therefore, PSM was first employed to match nine baseline clinical characteristics of patients at a 1:1 ratio. This balancing of intergroup differences ensured the comparability of baseline characteristics between the two patient groups, thereby minimizing the impact of confounding factors on the observed variables. After rigorous PSM to adjust for potential confounders, our findings demonstrated that the GnRH-a protocol was associated with significantly higher CLBR and CPR per woman compared with the GnRH-A protocol.

A comparison of secondary indicators between the GnRH-a and GnRH-A protocols revealed that the total Gn dosage was comparable between the two groups, whereas the GnRH-a protocol was associated with a longer stimulation duration and significantly higher serum estradiol levels on the trigger day relative to the GnRH-A protocol. Previous studies have indicated no differences in oocyte morphology between the two protocols (Murber *et al.* 2009, Pu *et al.* 2011, Borini & Bianchi 2012). Consistent with these prior randomized data, our current study found no significant differences in oocyte count or embryo quality between the groups, suggesting that the GnRH-A regimen may not exert a distinct effect on ovarian response.

Previous studies have demonstrated suboptimal pregnancy outcomes with fresh embryo transfer in the GnRH-A protocol. A retrospective study involving 1,119 fresh embryo transfer IVF cycles reported significantly lower implantation rates and CPRs in the GnRH-A protocol compared with the GnRH-a protocol (Xia & Zheng 2021). Other retrospective studies have shown that patients undergoing fresh embryo transfer with the GnRH-A protocol had significantly lower CPRs and live birth rates than those with frozen embryo transfer (Bahceci *et al.* 2009, Xu *et al.* 2018, Seyedoshohadaei *et al.* 2022). In our present study, after adjusting for confounding factors using multi-factor logistic regression analysis, the cumulative live birth rate (CLBR) in the GnRH-A protocol remained significantly lower than in the GnRH-a protocol. We also found that the GnRH-A protocol was associated with significantly lower CPRs, implantation rates, and live birth rates per transfer cycle in fresh embryo transfer cycles compared with the GnRH-a protocol. Notably, differences in clinical outcomes between the two protocols were less pronounced in frozen embryo transfer cycles. This may be attributed to the fact that endometrial preparation for frozen embryo transfer is not directly influenced by the COS protocol used during ovarian stimulation; instead, endometrial receptivity is primarily determined by the hormone replacement therapy employed for endometrial preparation. Overall, our data confirm that the GnRH-A protocol results in lower CLBR and CPR per woman compared with the GnRH-a protocol.

Endometrial thickness is recognized as a predictor of reproductive outcomes in both fresh and frozen embryo transfer cycles (Zhang *et al.* 2018). Orvieto *et al.* demonstrated that the GnRH-A group exhibited significantly thinner endometrium compared with the GnRH-a group (Orvieto *et al.* 2008). Ruan *et al.* found that GnRH-a, but not GnRH-A, partially restored physiological endometrial secretion and improved uterine receptivity in mice (Ruan *et al.* 2006). In addition, a comparative proteomic analysis revealed that endometrial receptivity is more severely impaired by GnRH-A than by GnRH-a protocols (Chen *et al.* 2019). Consistent with these findings, our present results showed that endometrial thickness on the day of fresh embryo transfer was significantly lower in the GnRH-A protocol than in the GnRH-a protocol. These observations align with previous studies reporting detrimental effects of GnRH-A on endometrial receptivity and thickness (Chen *et al.* 2020, Xu *et al.* 2022). Integrating previous research with our current findings, we speculate that the reduced endometrial thickness and impaired endometrial receptivity associated with the GnRH-A protocol may contribute to its inferior clinical outcomes.

Previous studies have suggested that GnRH-A may interfere with the regulation of endometrial genes critical for implantation, such as HOXA10 and c-kit (Chen *et al.* 2020). Its rapid action in inhibiting GnRH receptors may disrupt the delicate hormonal balance required for proper endometrial development and receptivity. However, the mechanism underlying the observed differences in endometrial receptivity between GnRH-a and GnRH-A protocols remains incompletely understood. Endometrial decidualization refers to the differentiation of endometrial stromal cells (ESCs) into specialized decidual stromal cells (DSCs) under the regulation of estrogen and progesterone (E2/P4) (Okada *et al.* 2018). As a prerequisite for mammalian embryo implantation, this process primarily occurs during the late secretory (luteal) phase of the menstrual cycle or early pregnancy. DSCs secrete nutritive factors (e.g., PRL, IGFBP-1) to provide energy and nutrients to the embryo. Mesenchymal-epithelial transition (MET) describes the cellular reprogramming of mesenchymal-like cells (spindle-shaped, low adhesion, high migration) to acquire epithelial characteristics (polygonal morphology, polarity, tight junctions) (Crha *et al.* 2019, Owusu-Akyaw *et al.* 2019, Guarino 2025). Within the endometrium, the core function of MET is to transform loose, migratory ESCs into epithelial-like decidual cells – establishing intercellular tight junctions (e.g., upregulated E-cadherin) and polarity to support embryo-endometrium anchoring (Owusu-Akyaw *et al.* 2019, Yang *et al.* 2023). For instance, downregulated E-cadherin (a marker of impaired MET) prevents stromal cells from forming epithelial-like structures, reducing IGFBP-1 and PRL secretion and ultimately leading to decidualization failure (Wu *et al.* 2017, Yang *et al.* 2017). Clinically, endometrial tissues from patients with recurrent implantation failure exhibit significantly lower expression of MET markers (E-cadherin, Occludin) and decidualization markers (IGFBP-1, HOXA10) than those from normal controls, suggesting these abnormalities are key contributors to implantation failure (Yang *et al.* 2017, Shen *et al.* 2020). Previous studies have shown that the cellular morphological changes observed during decidualization are consistent with the MET process and are regulated by ovarian hormones (Ramathal *et al.* 2010, Xiong *et al.* 2024). In our study, the expression of the epithelial marker E-cadherin was higher, while that of the mesenchymal marker Vimentin was lower, in secretory-phase endometrial tissues from the GnRH-A treatment group compared with the GnRH-a group. Our *in vitro* experiments further confirmed this observation: GnRH-A inhibits the decidualization of HESCs, which is specifically manifested by decreased expression of PRL and IGFBP1, and this effect is mediated by reducing MET formation.

CLBR is the primary indicator used in assisted reproduction to evaluate the success of IVF (Yang *et al.* 2022). CPR per woman refers to the overall probability of achieving a clinical pregnancy using all embryos generated from a single oocyte retrieval cycle, including those transferred in the fresh cycle and subsequent transfers of cryopreserved-thawed embryos (Sun *et al.* 2024). In the present study, our data confirm that the GnRH-A protocol is associated with lower per-woman CPR and CLBR compared with the GnRH-a protocol. This outcome may be attributed to the significantly lower per-cycle live birth rate and CPR observed in the GnRH-A protocol during fresh embryo transfer cycles relative to the GnRH-a protocol. A decline in the per-cycle pregnancy rate during fresh cycles essentially stems from two key factors: a reduction in immediate pregnancy opportunities and a decrease in the number of viable embryos available for cryopreservation (and subsequent thawing). In contrast, the core role of frozen-thawed embryo transfer (FET) cycles is to utilize cryopreserved embryo reserves to supplement pregnancy chances. However, the per-cycle pregnancy rate of FET cycles remains relatively stable, and their effectiveness inherently depends on the quantity and quality of embryos preserved from fresh cycles. Consequently, if the decline in ‘embryo yield’ and ‘immediate pregnancy contribution’ from fresh cycles in the GnRH-A protocol exceeds the compensatory capacity of FET cycles, the overall cumulative CPR and cumulative live birth rate will inevitably decrease – even if the per-cycle success rate of FET cycles remains unchanged.

This study also has certain limitations. Specifically, the number of fresh embryo transfer cycles in the GnRH-A group was significantly lower than that in the GnRH-a group (97 vs 308). We analyzed the potential contributing factors as follows: i) Endometrial factors: GnRH-A prevents premature LH surges by competitively binding to and directly inhibiting LH receptors in a rapid manner. While effective for LH surge suppression, this mechanism may disrupt the normal hormonal balance required for endometrial differentiation. Consequently, it is hypothesized that the GnRH-A group had a substantially higher number of cycles where fresh embryo transfer was canceled due to ‘endometrial-embryo asynchrony or insufficient endometrial thickness’ compared with the GnRH-a group – directly contributing to the reduced number of final fresh transfer cycles in the GnRH-A group. ii) Risk management of OHSS: Although the overall incidence of OHSS in the GnRH-A protocol is lower than that in the GnRH-a protocol, more stringent risk control measures are implemented for patients with ovarian hyperresponse (e.g. patients with polycystic ovary syndrome, young patients, or those with a high AFC). Notably, ‘canceling fresh embryo transfer’ is a critical strategy to prevent moderate-to-severe OHSS, as pregnancy-induced elevation of hCG can exacerbate OHSS. This proactive risk mitigation further widened the gap in the number of fresh embryo transfer cycles between the two groups. iii) Clinical decision-making based on prior evidence: a large body of previous research has consistently demonstrated that the pregnancy outcomes of fresh embryo transfer following the GnRH-A protocol are significantly inferior to those of the GnRH-a protocol. In line with this evidence, our reproductive center adopts a more conservative approach when considering fresh embryo transfer in the GnRH-A protocol. Specifically, if any clinical or laboratory indicator fails to meet predefined transfer criteria (e.g., suboptimal endometrial morphology, marginal hormonal profiles), the decision to perform whole-embryo cryopreservation is prioritized over fresh transfer. This cautious strategy further reduced the number of fresh transfer cycles in the GnRH-A group.

Our study provides additional evidence that the GnRH-A protocol is associated with lower per-cycle CPR and live birth rate in fresh ET, as well as reduced per-woman CPR and CLBR, compared with the GnRH-a protocol. This difference is likely attributed to the adverse effect of GnRH-A on endometrial receptivity. However, given the potential for selection bias and other inherent limitations of retrospective studies, further randomized controlled trials are required to validate our findings. Clinicians should carefully weigh the potential risks and benefits of different COS protocols when formulating individualized treatment plans for patients.

## Supplementary materials



## Declaration of interest

The authors declare that there is no conflict of interest that could be perceived as prejudicing the impartiality of the research reported.

## Funding

This project was supported by the National Natural Science Foundation of China (No. 82071722 to W Xiong 2020) and the ‘Fertility Research Program of Young and Middle-aged Physicians Basic Research in 2022’ (No. BJHPA-2022-SHZHYXZHQNYJ-JCH-001) of Beijing Health Promotion Association, donated by Merck Serono Co., Ltd.

## Author contribution statement

XC performed the experiments. HD analyzed and interpreted the data. WX and YL designed the study, originally obtained funding, and drafted the manuscript. All authors approved the final manuscript.
