# Supplementary material for: GnRH agonist improves CLBR after one IVF cycle: a propensity score-matched and molecular mechanism study
Source: Reprod Fertil. 2025 Dec 9;6(4):e250045. doi: 10.1530/RAF-25-0045 (PMC12697241; doi:10.1530/RAF-25-0045)
Supplement: Supplementary file 1 [file supplementary_materials.pdf]

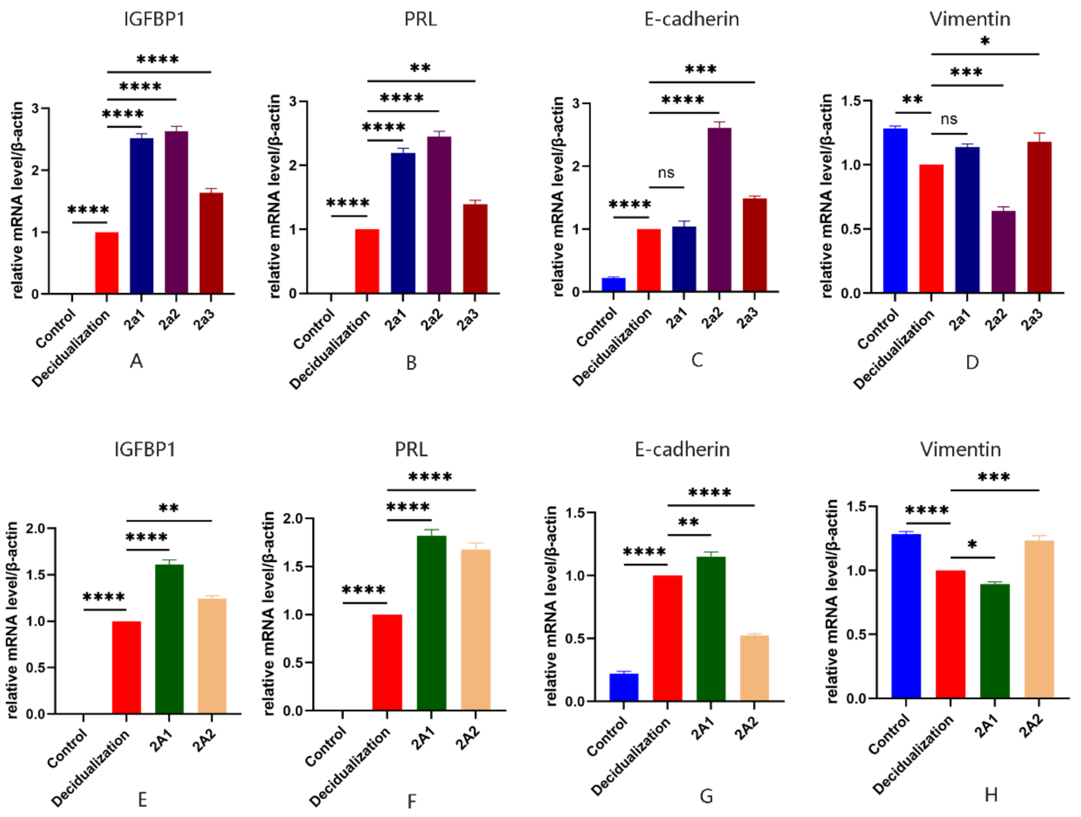

Supplement figure 1. In order to determine the optimal action concentration of GnRH-a and GnRH-A, the expression of the decidualization markers (PRL and IGFBP1) and MET markers (E-cadherin and Vimentin) were detected. A-D, ESCs were divided into five groups (control, decidualization, 2a1, 2a2 and 2a3) in vitro. The mRNA levels of IGFBP1, PRL, E-cadherin and vimentin were examined by qRT-PCR. E-H, ESCs were divided into four groups (control, decidualization, 2A1 and 2A2) in vitro. The mRNA levels of IGFBP1, PRL, E-cadherin and vimentin were examined by qRT-PCR. (ns=no significant; 2a1, 2a2 and 2a3 means that the treatment days of GnRH-a are two days and the concentration is 5.00×10^{-10}mol/L, 9.33×10^{-10}mol/L and 2.04×10^{-10}mol/L respectively; 2A1 and 2A2 means that the treatment days of GnRH-A are two days and the concentration is3.55×10^{-10}mol/L and 8.87×10^{-10}mol/L respectively; *P<0.05, **P<0.01, ***P<0.001, ****P<0.0001)

Supplementary Table 1. Primer characteristics

Names	Sequences
IGFBP1-F	TTTTACCTGCCAAACTGCAACA
IGFBP1-R	CCCATTCCAAGGGTAGACGC
PRL-F	GGAGCAAGCCCAACAGATGAA
PRL-R	GGCTCATTCCAGGATCGCAAT
E-cadherin-F	ATTTTTCCCTCGACACCCGAT
E-cadherin-R	TCCCAGGCGTAGACCAAGA
Vimentin-F	AGTCCACTGAGTACCGGAGAC
Vimentin-R	CATTTCACGCATCTGGCGTTC
β-actin-F	CACCATTGGCAATGAGCGGTTC
β-actin-R	AGGTCTTTGCGGATGTCCACGT

Supplementary Table 2. Commercial sources and characteristics of antibodies used.

Antibody	Dilution			Isotype	Product Num/ Manufacture	Location
	IHC	WB	Elisa			
E-cadherin	1:1600	-	-	Rabbit IgG	ab40772/Abcam	U.S.
	-	1:1000	-	Rabbit IgG	WY0B /proteintech	China
Vimentin	1:300	1:2000	-	Rabbit IgG	#AF7031 /Affinity	U.S.
GAPDH	-	1:200000	-	Rabbit IgG	/proteintech	China
IgG	1:2000	1:5000	1:4000	Goat Anti-Rabbit IgG	ab150077/Abcam	U.S.
